# Frontal White Matter Volume Is Associated with Brain Enlargement and Higher Structural Connectivity in Anthropoid Primates

**DOI:** 10.1371/journal.pone.0009123

**Published:** 2010-02-09

**Authors:** Jeroen Bert Smaers, Axel Schleicher, Karl Zilles, Lucio Vinicius

**Affiliations:** 1 Leverhulme Centre for Human Evolutionary Studies, Department of Biological Anthropology, University of Cambridge, Cambridge, United Kingdom; 2 Cécile and Oskar Vogt Institute for Brain Research, Heinrich-Heine-University, Düsseldorf, Germany; University of Lethbridge, Canada

## Abstract

Previous research has indicated the importance of the frontal lobe and its ‘executive’ connections to other brain structures as crucial in explaining primate neocortical adaptations. However, a representative sample of volumetric measurements of frontal connective tissue (white matter) has not been available. In this study, we present new volumetric measurements of white and grey matter in the frontal and non-frontal neocortical lobes from 18 anthropoid species. We analyze this data in the context of existing theories of neocortex, frontal lobe and white versus grey matter hyperscaling. Results indicate that the ‘universal scaling law’ of neocortical white to grey matter applies separately for frontal and non-frontal lobes; that hyperscaling of both neocortex and frontal lobe to rest of brain is mainly due to frontal white matter; and that changes in frontal (but not non-frontal) white matter volume are associated with changes in rest of brain and basal ganglia, a group of subcortical nuclei functionally linked to ‘executive control’. Results suggest a central role for frontal white matter in explaining neocortex and frontal lobe hyperscaling, brain size variation and higher neural structural connectivity in anthropoids.

## Introduction

Previous research has shown that the neocortex (or ‘neopallium’) has played a central role in the evolution of brain size and architecture in primates and other mammals [1]. Primates vary greatly in terms of overall brain size, and most of the variation among species is explained by the relative size increase (or ‘hyperscaling’) of the neopallium in larger primate brains [2], [3]. Overall, evolutionary changes in the size of neopallium and other brain structures are expressed in three ways: (1) a disproportionate increase of white versus grey matter; (2) a disproportionate increase of particular structures or areas; and (3) evolutionary changes in neural circuits of interconnecting structures.

The neopallium comprises both neocortical grey matter (consisting of neural cell bodies, their dendrites and parts of their axons as well as glial cells, and responsible for synaptic activity and the processing of information) and white matter (mostly consisting of bundles of myelinated or non-myelinated axons connecting cortical regions (i.e. grey matter) to each other and to subcortical structures). Barton and Harvey [3] found that the hyperscaling of the neopallium in primates is entirely due to white matter, with grey matter scaling only isometrically (in direct proportion with the rest of the brain). Zhang & Sejnowski [4] have shown that the hyperscaling of the neopallial white matter relative to grey matter occurs in all mammals, generalizing the finding of Allman [1] who had already described a scaling factor of about 4/3 between white and grey matter in primates. In larger brains, due to the larger distance between neurons and the length of the axons connecting them, longer axons need to be thicker and more heavily myelinated in order to maintain optimal conduction times, causing white matter volume to hyperscale with cortical volume [2], [4], [5], [6], [7]. In summary, because white matter consists of connective fibers linking neocortical regions to each other and to subcortical structures, it is of critical importance when investigating connectivity in the brain.

The evolution of primate brain size has also been associated with changes in the relative size of particular brain components. In primates, the neopallial frontal lobe (meaning frontal grey plus frontal white matter) has received particular attention in debates over the neuroanatomical foundations of ‘intelligence’. The frontal lobe has been linked to ‘executive functions’ [8], ‘theory of mind’ abilities [9], [10], [11] and ‘higher cognitive functions’ [12]. Semendeferi et al. [13], [14] collected frontal lobe volumetric data for humans, five other apes and two monkeys and showed that the frontal lobe hyperscales with the rest of neopallium; thus, a larger brain tends to exhibit a relatively larger neopallium, which in turn exhibits a relatively larger frontal lobe. Therefore, these studies suggest that the evolution of larger brains in primates is associated with a disproportionate enlargement in the frontal lobe, the neocortical region responsible for the processing of higher cognitive functions. The roles of white matter volume and frontal lobe volume in brain size variation suggest that they may have a joint effect in explaining variation in brain size. If the primate frontal lobe performs a ‘central executive’ or integrative function due to its neural connections to other brain regions, its relative enlargement is expected to be caused by a specific increase in the volume of its connective tissue (i.e. white matter). Schoenemann et al. [15] have demonstrated that both the total size of prefrontal cortex and specifically prefrontal white matter are exceptionally enlarged in humans in comparison with other hominoids, whereas prefrontal grey matter showed no significant difference. Their results have, however, been questioned by Sherwood et al [16] who pointed to the difficulties associated with identifying the prefrontal on the basis of gross anatomical rather than cytoarchitectonic features.

Because the frontal lobe is considered to exert executive type control over other structures it is expected to show significant connectivity to other brain structures. Brain structures are commonly known to work as part of functional systems in which interconnected structures work together in processing particular types of information. The ‘mosaic’ hypothesis of brain evolution suggests that selective pressures act directly on these interconnected neural circuits rather than on single brain areas [3] suggesting investigations into primate brain evolution would benefit from focussing on neural circuits of interconnected brain structures rather than on single structures in isolation [17]. Semendeferi et al. [14] indicated that since the total size of the human frontal lobe is not exceptionally large for great ape standards (i.e. the human frontal lobe is as large as predicted for a great ape with our brain size), our higher cognitive functions might be explained by the possibility that neural circuits are more richly interconnected within the frontal sectors themselves and/or between those sectors and other brain regions. They were, however, unable to test this hypothesis using their database. Of particular importance in the context of the frontal lobe is the cortico-basal ganglia neural circuit that interconnects mainly prefrontal areas to the basal ganglia [18], [19], [20], [21], [22], [23], [24], [25], [26] and is functionally associated to elements of ‘executive control’: control and selection of actions [27]; guidance of reward related behaviour [28], [29]; conscious, goal-directed behaviour [30], [31], [32]; and learning of complex motor skill behaviour [33], [34], [35].

The studies above have pointed to white matter volume and frontal lobe volume in the neopallium as crucial to the understanding of brain size variation in primates and suggest two areas of further improvement: First, it would be important to investigate the specific contribution of the frontal white, non-frontal white, frontal grey and non-frontal grey to brain size variation, which has not yet been possible due to the non-availability of data. Second, it would be relevant to empirically test the hypothesis that frontal (but not non-frontal) white matter volume across species should correlate with the volume of structures such as the basal ganglia.

In this study, new volumetric measurements of the neopallium and neopallial frontal, non-frontal, white and grey matter were obtained for 18 anthropoid primates (excluding humans) (see Table 1). The advantage of the current dataset is twofold: first, volumes are available for frontal grey, frontal white, non-frontal grey and non-frontal white matter in all species (previous studies either combined grey and white matter into one volumetric measurement [13], [14] or measured only the former [36]); Second, measurements were taken from the same specimens for which volumetric data are available for a wide range of other brain structures including the basal ganglia [37]. In the following, we investigate whether the neopallial scaling law between white and grey matter also applies separately for the frontal and non-frontal lobe; whether the hyperscaling of the frontal lobe is due to white or grey matter, or both; and how frontal (and non-frontal) grey and white matter scale to the basal ganglia and to the rest of the brain.

**Table 1:** Volumetric measurements of neopallium and frontal lobe white and grey matter.

Species	Specimen #	Current study						Fr/Neo (%)			Stephan (unpublished)		
		N	Nw	Ng	FR	FRw	FRg	Current study	Semendeferi et al. 1997	Semendeferi et al. 2002	N	Brain	BG
*Pan troglodytes*	280	321677	130114	191563	117753	53062	64691	36.6	35.9	35.4(±1.9)	331912	444981	15890
*Pan paniscus*	yn860137	235469	76921	158548	79632	29100	50532	33.8		34.7(±0.6)		378400	
*Gorilla gorilla*	375	307550	98839	208711	108754	35905	72848	35.4	32.4	35.0and36.9	307299	434363	15714
*Pongo pygm.*	297	327005	143847	183158	123641	59201	64440	37.8	35.3	37.6(±1.1)		424700	
*Hylobates lar*	1203	66499	19891	46608	19249	6067	13182	28.9	31.1	29.4(±1.8)	65735	98359	5495
*Papio anubis*	97	130886	51365	79521	38136	15087	23049	29.1			132254	184363	8230
*Cercoceb. alb.*	242	73005	25173	47832	21386	7568	13818	29.3			73496	106660	5018
*Cercopith. Mit.*	261	49500	15693	33807	15273	5057	10215	30.9			49933	72394	
*Cercopith. asc.*	219	40393	12121	28273	10261	3143	7119	25.4			41459	59363	3000
*Erythroceb. p.*	1341	70004	24210	45794	16641	5878	10763	23.8			69118	93726	
*Miopith. tal.*	1171	26918	7181	19738	7083	1895	5188	26.3			27175	39672	
*Nasalis larv.*	1365	42022	12037	29985	10997	2941	8055	26.2			41521	62017	3024
*Procol. bad.*	213	51273	14651	36622	15015	4400	10615	29.3			51420	75965	4025
*Alouatta sen.*	1184	27357	7535	19822	8174	2753	5421	29.9			27385	45174	
*Ateles geoffroyi*	1000	69323	16627	52696	22151	5962	16190	32.0			69807	102703	5684
*Lagothr.lagotr.*	1571	60026	19202	40824	17017	5872	11145	28.3			58994	88156	5521
*Pith. monachus*	1180	20862	6261	14601	5482	1654	3828	26.3			20659	32819	
*Cebus albifrons*	1200	52450	18212	34237	14694	5523	9170	28.0		29.6and31.5	52941	77027	4193

Species #: identification number of the individual used in the collection; N: neopallium; w: white matter; g: grey matter; FR: frontal neopallial lobe; Brain: total brain volume; BG: basal ganglia. The ratio of frontal lobe relative to neopallium are indicated for the current study and for the analyses done on different individuals by Semendeferi et al (1997) and Semendeferi et al. (2002). Basal ganglia is measured as the sum of its two largest constituent parts (striatum and pallidum) that are also responsible for (respectively) the primary input from neopallium and primary output to other brain structures. Volumetric data for substantia nigra and subthalamic nucleus (the two other, but significantly smaller, constituent parts of the basal ganglia) are not available for the individuals used in the current analysis. Data from Stephan (unpublished) comprises the individual-specific data underlying the data presented in Stephan et al. [37] and was made available by H. Frahm. Data on brain size from *Pan paniscus* and *Pongo pygmaeus*, however, comes from Macleod et al. [61].

## Results

### Scaling Law of White Versus Grey Matter

In terms of the scaling relationship between white and grey matter, results of PGLS regressions indicate a scaling coefficient or regression slope of *b* = 1.180 (95% C.I. = 1.062–1.298, α = 15.5, *N* = 18) when considering the neopallium as a whole. For the frontal lobe, the slope is *b* = 1.180 (95% C.I. = 1.082–1.278, α = 15.5, *N* = 18) and in the non-frontal, *b* = 1.170 (95% C.I. = 1.052–1.288, α = 15.5, *N* = 18).

### Hyperscaling of Neocortex, White Matter and Frontal Lobe

Neocortical white matter hyperscales with the rest of the brain (calculated as brain volume minus neopallium volume), rendering a slope of *b* = 1.210 (95% C.I. = 1.034–1.386, α = 15.5, *N* = 18). Overall neocortical volume and neocortical grey matter, however, scale isometrically to the rest of the brain (*b* = 1.090, 95% C.I. = 0.972–1.208, α = 15.5, *N* = 18; *b* = 1.030, 95% C.I. = 0.932–1.128, α = 15.5, *N* = 18).

When the neopallium is divided into frontal and non-frontal lobe, frontal lobe volumes hyperscale with the rest of the brain (brain minus frontal lobe; *b* = 1.190, 95% C.I. = 1.151–1.229, α = 15.5, *N* = 18; Table 2). In contrast, the non-frontal lobe scales isometrically to the rest of the brain (brain minus non-frontal lobe; *b* = 0.990, 95% C.I. = 0.951-1.029, α = 6.2, *N* = 18). Furthermore, both frontal lobe white and grey matter significantly hyperscale with the rest of the brain (brain minus frontal lobe; *b* = 1.330, 95% C.I. = 1.232–1.428, α = 15.5, *N* = 18; *b* = 1.110, 95% C.I. = 1.051–1.169, α = 15.5, *N* = 18). For the non-frontal lobe, white matter hyperscales while grey matter hyposcales to the rest of the brain (brain minus frontal lobe; *b* = 1.120, 95% C.I. = 1.002–1.238, α = 4.77, *N* = 18; *b* = 0.930, 95% C.I. = 0.891–0.969, α = 7.04, *N* = 18).

**Table 2:** Scaling of neocortical substructures to rest of brain.

Y	X	slope	95% C.I.	
			Min.	Max.
N	Brain minus N	1.09	0.97	1.21
Nw	Brain minus N	1.21	1.03	1.39
Ng	Brain minus N	1.03	0.93	1.13
FR	Brain minus Fr	1.19	1.15	1.23
FRw	Brain minus Fr	1.33	1.23	1.43
FRg	Brain minus Fr	1.11	1.05	1.17
NF	Brain minus NF	0.99	0.95	1.03
NFw	Brain minus NF	1.12	1.00	1.24
NFg	Brain minus NF	0.93	0.89	0.97

NF: non-frontal neopallial lobe; for other abbreviations, see table 1. Results of a PGLS regression of neocortical structures (Y) on measures of ‘rest of brain’ (X). Values indicate the slope and its 95% confidence interval (‘C.I.’).

### Correlations with Basal Ganglia and Rest of Brain

A partial regression of both whole frontal lobe volume and frontal white matter with basal ganglia (controlling for the effects of brain minus frontal lobe and basal ganglia) yield significant positive associations (*b* = 0.260, 95% C.I. = 0.064–0.456, *R^2^* = 0.442, α = 15.5, *N* = 11; *b* = 0.800, 95% C.I. = −0.004–1.604, *R^2^* = 0.321, α = 2.1, *N* = 10), while no such association exists with whole non-frontal lobe volume or non-frontal white matter (*b* = 0.100, 95% C.I. = −0.214–0.414, *R^2^* = 0.044, α = 15.5, *N* = 11; *b* = −0.020, 95% C.I. = −1.137–1.097, *R^2^* = 0.000, α = 6.240, *N* = 11) (Table 3). Furthermore, a partial regression of both whole frontal lobe volume and frontal white matter indicate significant associations with the rest of the brain (brain minus neopallium) when the effects of the non-frontal lobe are controlled for (*b* = 0.360, 95% C.I. = 0.086–0.634, *R^2^* = 0.283, α = 15.5, *N* = 18; *b* = 0.730, 95% C.I. = 0.201–1.259, *R^2^* = 0.338, α = 15.5, *N* = 16), while whole non-frontal lobe volume and non-frontal white matter indicate no such association (after controlling for the effects of frontal lobe volume: *b* = 0.020, 95% C.I. = −0.313–0.353, *R^2^* = 0.001, α = 15.5, *N* = 18; *b* = −0.360, 95% C.I. = −1.007–0.287, *R^2^* = 0.069, α = 2.1, *N* = 18) (Table 3).

**Table 3:** Correlations between neocortical substructures and basal ganglia and rest of brain.

X	Control	Y		
		FR	FRw	FRg
BG	Brain minus FR and BG	44.2**	32.1*	2.5
Brain minus N	NF	28.3*	33.8**	40.0**
		NF	NFw	NFg
BG	Brain minus NF and BG	4.4	0.0	9.3
Brain minus N	FR	0.1	6.9	7.7

For abbreviations see tables 1 and 2. Values indicate partial R^2^ values of a PGLS regression between basal ganglia and rest of brain (‘Brain minus N’) with frontal and non-frontal lobe white and grey matter. Partial correlations were computed by using the residuals of each variable to the control variable (‘control’).

When considering grey matter, frontal grey matter correlates with the rest of the brain (after controlling for the effects of non-frontal lobe size: *b* = 0.480, 95% C.I. = 0.186–0.774, *R^2^* = 0.400, α = 15.5, *N* = 18) but not with basal ganglia (*b* = 0.090, 95% C.I. = −0.302–0.482, *R^2^* = 0.025, α = 1.1, *N* = 11), while non-frontal grey matter does not indicate associations to either the rest of the brain (after controlling for the effects of frontal lobe size) or basal ganglia (*b* = 0.020, 95% C.I. = −0.114–0.514, *R^2^* = 0.093, α = 15.5, *N* = 18; *b* = 0.090, 95% C.I. = −.106–0.286, *R^2^* = 0.077, α = 15.5, *N* = 18).

## Discussion

The analyses presented here corroborate current scaling models of connectivity in brains of varying size [38], [39]. Using new data for anthropoid primates, we estimated a scaling parameter close to the suggested 4/3 [1], [4] between the entire neopallial white and grey matter, and also between white and grey matter separately in the frontal and the non-frontal lobe. Although the scaling parameter indicated in the current study is slightly lower than the suggested 4/3 (around 3.5/3), results show that the hyperscaling of white matter in the neopallium is consistent in both the frontal and non-frontal lobe and thus not due to a disproportionate effect in either lobe. Furthermore, results indicate that the hyperscaling of neopallium to the rest of the brain is mainly due to the effect of white matter, corroborating previous research [3].

Considering the possible separate contribution of the frontal and non-frontal lobe to brain size variation in anthropoids, our results are in line with studies indicating that the frontal lobe hyperscales with the rest of the brain [13], [14], and additionally show that the non-frontal lobe scales in direct proportion to the rest of the brain suggesting that the hyperscaling of the neocortex is due to the effects of the frontal lobe only. A combined effect of frontal lobe and white matter was also identified. The hyperscaling of the frontal lobe with the rest of the brain is mainly due to the effect of white matter (*b* = 1.33 for white matter; *b* = 1.11 for grey matter) and frontal white matter (but not non-frontal white matter) is found to correlate significantly with the rest of the brain (variation in the volume of frontal white matter explains 33.8% of the variation in the size of the rest of the brain after controlling for the effects of non-frontal lobe size, while volume changes in non-frontal white matter explain only 6.9% after controlling for the effects of frontal lobe size). Together, these results suggest that frontal white matter can be considered a principal component in explaining variation in brain size.

We also found a significant and exclusive correlation between variation in frontal white matter volume and variation in basal ganglia volume. Variation in frontal white matter explains 32.8% of the variation in the size of basal ganglia while non-frontal white matter explains 0%. This result provides comparative evidence for the cortico-basal ganglia neural circuit [19], [21] that is thought to underlie ‘executive functions’ such as learning [33], [34], [35] and suggests that it is mainly the frontal lobe connecting to the basal ganglia (making it a ‘frontal cortico-basal ganglia’ neural circuit) corroborating neuroanatomical studies showing that the main input into the basal ganglia comes from prefrontal areas [20], [21], [22].

We have presented novel volumetric measurements of frontal and non-frontal white and grey matter in 18 anthropoid primates and indicated that frontal white matter is a principal component in explaining variation in brain size and hyperscaling or relative enlargement of the neocortex in anthropoid evolution. We have also provided comparative neuroanatomical evidence for the presence of a frontal cortico-basal ganglia neural circuit (associated to ‘higher executive functions’ such as learning) by showing that only variation in frontal white matter size explains variation in the size of the basal ganglia. This finding implies that frontal white matter is at the heart of increased structural connectivity associated to brain enlargement and higher cognitive capacities. Future studies should focus on further delineation of frontal substructures based on cytoarchitectonic and including more taxa into the analysis (possibly going beyond the delineation of prefrontal areas proposed by Semendeferi et al.[40] and overcoming the contention of demarcation based on gross anatomical structures by Schoenemann et al.[15]).

## Materials and Methods

### Brains and Volumetric Measurements

We examined brains from 18 anthropoid species, all of which are housed at the C. & O. Vogt Institute for Brain Research (Heinrich-Heine-University, Düsseldorf, Germany). The sample used in this study is part of a widely used collection in primate comparative neuroanatomy [3], [41], [42]. The collection consists of serial, Nissl-stained brain sections. The traditional procedure to delineate volumes from histological sections was used [Cavalieri method: 43,44]. Systematic random samples from each brain were taken, the position of the first section was chosen randomly and subsequent sections were chosen based on a regular sampling interval. 20 or more sections per brain (indicated by Zilles et al. [45] as a sufficient number to determine the volume of extremely irregular formed bodies) were used and digitized with a standard office flatbed scanner at 800dpi. Surface area was computed on each section and volumes were estimated by summing the areas across sections and multiplying by slice thickness. Left hemisphere data was computed and inferences were made for both hemispheres. Overall neopallial frontal lobe volumes of white and grey matter were computed and non-frontal volumes were defined accordingly. When a regularly spaced section was missing or of insufficient quality, an adjacent section was used. Shrinkage ratios were available for all specimens used and applied to estimate fresh volumes.

### Demarcation of Boundaries

#### Neopallium

The boundary between white matter and all ventromedial non-neocortical structures was manually drawn making use of a brain atlas [46]. Neocortical volumes computed in this study were compared to the volumes of the same specimens as measured by Stephan et al. [37] (unpublished specimen specific data provided by Dr. H. Frahm). The average discrepancy between the two measures is 0.29% with a standard deviation of 1.36%. The highest difference was found for *Pan troglodytes* (3.08%).

#### Frontal lobe

The boundary between the frontal and non-frontal lobe was delineated using cytoarchitectonic criteria. On its posterior end, the frontal lobe is defined as that part of the neopallium anterior to the boundary between the primary motor cortex (area 4) and the somatosensory cortex (area 3) [47]. Apart from the boundary of the primary visual cortex (area V1) in primates, this is the most easily recognizable and reliable cytoarchitectonic border in the neopallium [48], and has also been identified electrophysiologically in various primate species [49], [50], [51]. In anthropoid primates, the motor-somatosensory border is located in the fundus of the central sulcus [47], [52], [53], [54], [55]. Six cytoarchitectonic features change on the motor-somatosensory border [e.g., 36,47] and were used in the current analysis (for area 4 and area 3 respectively): cell-shape (pyramidal and elongated Betz cells versus absence of Betz cells); cell density (low versus high); cell size (large versus small); granular layer IV (absent versus present); border between white and grey matter (diffuse versus sharp); lamination (diffuse versus sharp). The posterior border of the frontal lobe (the motor-somatosensory border) was prolonged in a straight line transversely to the most posterior segment of the ventromedial end of frontal lobe white matter. The demarcation then follows the boundary between the frontal lobe white matter and the medial non-neocortical areas excluded from the analysis until above the corpus callosum (which is considered the posterior limit of the frontal lobe white matter on the medial surface). Although Semendeferi et al. [13] and Semendeferi et al. [14] obtained frontal lobe measurements from different specimens using a different delineation procedure (based on sulcal patterns using MRI-images), the ratios of frontal lobe to overall neopallial volume as obtained in these studies correspond to those obtained in the current study (see appendix 1).

### Statistical Analysis

#### Phylogenetic controls

We used phylogenetically controlled generalized least squares analysis (PGLS) to compute comparative correlations between the relative sizes of brain structures across taxa. This procedure is preferred over the traditionally more popular independent contrasts (IC [56]) approach because it allows the assumed model of evolution to deviate from Brownian Motion by using a maximum likelihood estimate of a parameter (α) that indicates the strength of the evolutionary constraint [57]. This approach is more general than IC because its results are identical to IC results when the estimated alpha parameter is zero. The phylogeny was taken from Smith & Cheverud [58] and supplemented with data for *Pan paniscus* from Purvis [59]. PGLS analyses were executed in COMPARE 4.6b [60].

#### Measures of ‘relative’ brain structure co-evolution

The ‘relative’ sizes of brain structures were computed as residuals of a regression of a particular structure to the brain minus that structure. When the comparative correlation between the relative sizes of two brain structures was computed, a regression was computed using residuals of a regression of each structure to the brain minus both structures. E.g., for the comparative correlation between frontal lobe size and basal ganglia size, the independent variable constitutes the residuals of a regression of frontal lobe size to brain minus frontal lobe and basal ganglia size, the dependent variable comprises the residuals of a regression of basal ganglia size to brain minus frontal lobe and basal ganglia size. This approach ensures that two brain areas are always compared using the same control variable (so that the comparative platform is identical).
